# Repression of Tropolone Production and Induction of a *Burkholderia plantarii* Pseudo-Biofilm by Carot-4-en-9,10-diol, a Cell-to-Cell Signaling Disrupter Produced by *Trichoderma virens*

**DOI:** 10.1371/journal.pone.0078024

**Published:** 2013-11-04

**Authors:** Mengcen Wang, Makoto Hashimoto, Yasuyuki Hashidoko

**Affiliations:** Graduate School of Agriculture & Research Faculty of Agriculture, Hokkaido University, Sapporo, Japan; Ben-Gurion University of the Negev, Israel

## Abstract

**Background:**

The tropolone-tolerant *Trichoderma virens* PS1-7 is a biocontrol agent against *Burkholderia plantarii,* causative of rice seedling blight. When exposed to catechol, this fungus dose-dependently produced carot-4-en-9,10-diol, a sesquiterpene-type autoregulatory signal molecule that promotes self-conidiation of *T. virens* PS1-7 mycelia. It was, however, uncertain why *T. virens* PS1-7 attenuates the symptom development of the rice seedlings infested with *B. plantarii*.

**Methodology/Principal Findings:**

To reveal the antagonism by *T. virens* PS1-7 against *B. plantarii* leading to repression of tropolone production in a coculture system, bioassay-guided screening for active compounds from a 3-d culture of *T. virens* PS1-7 was conducted. As a result, carot-4-en-9,10-diol was identified and found to repress tropolone production of *B. plantarii* from 10 to 200 µM in a dose-dependent manner as well as attenuate virulence of *B. plantarii* on rice seedlings. Quantitative RT-PCR analysis revealed that transcriptional suppression of *N*-acyl-L-homoserine lactone synthase *plaI* in *B. plantarii* was the main mode of action by which carot-4-en-9,10-diol mediated the quorum quenching responsible for repression of tropolone production. In addition, the unique response of *B. plantarii* to carot-4-en-9,10-diol in the biofilm formed in the static culture system was also found. Although the initial stage of *B. plantarii* biofilm formation was induced by both tropolone and carot-4-en-9,10-diol, it was induced in different states. Moreover, the *B. plantarii* biofilm that was induced by carot-4-en-9,10-diol at the late stage showed defects not only in matrix structure but also cell viability.

**Conclusions/Significance:**

Our findings demonstrate that carot-4-en-9,10-diol released by *T. virens* PS1-7 acts as an interkingdom cell-to-cell signaling molecule against *B. plantarii* to repress tropolone production and induces pseudo-biofilm to the cells. This observation also led to another discovery that tropolone is an autoregulatory cell-to-cell signaling molecule of *B. plantarii* that induces a functional biofilm other than a simple *B. plantarii* virulence factor.

## Introduction

*Burkholderia plantarii*, a rice bacterial pathogen, produces tropolone as a phytotoxin and a virulence factor to cause seedling blight. Rice seedlings exposed to tropolone typically exhibit stunting as a blight symptom similar to the rice seedlings that have been infested with *B. plantarii* [1], [2]. In order to suppress this disease, biocontrol agents were selected that were catechol-resistant microbial from rice rhizosphere, and *Trichoderma virens* PS1-7 was found to be a marked competitor of pathogenic *B. plantarii*. The antagonistic effects exerted by *Trichoderma virens* PS1-7 against *B. plantarii* were found to be a dominant contribution to the repression of tropolone production in *B. plantarii* and protected rice seedlings inoculated with it [3].

From the perspective of cell-to-cell signaling, antagonism and mutualism in the microbial ecosystem indicate competitive and cooperative interaction regulated by chemical signaling molecules [4]–[7]. In the bacterial intraspecies cooperation, AHLs (*N-*acyl homoserine lactones) known as major quorum sensing (QS) signals were produced in many proteobacteria and functioned to coordinate intraspecies group-based behaviors via multicellular cell-to-cell signaling [8]. In addition, cell-to-cell signaling among living creatures were also reported in interspecies and even interkingdom interactions involving a wide array of chemical signaling molecules in a complex manner, including interaction between eubacteria and plants [5], [9], [10].

One pioneer study of interkingdom cell-to-cell signal communication from the plant-side has been done in interaction between a γ-proteobacterium *Serratia liquefaciens* and a red marine algae *Delisea pulchra* in marine ecosystem. *D. pulchra* produced two furanones that interfere in AHL-mediated cellular processes of the epiphytic bacterium [11]. The AHL-mimics prevented LuxR protein to bind to promoter region of QS-regulated genes and blocked expression of QS-regulated genes in *Vibrio harbeyi* cells [12]. Conversely, quormone mimics secreted from plant roots were first found in the seedling of pea (*Pisum sativum*) [13], and L-canavanine from the roots of alfalfa (*Medicago sativa*) was first characterized as a QS-interfering compound in terrestrial ecosystems [14]. To date, quorum quenching (QQ) by plants has extensively been studied, but chemical compounds identified as quorum quenchers are limited to few numbers [15]. As an interkingdom communication between fungi and eubacteria, *Candida albicans* isolated from the lungs of patients with cystic fibrosis reduced virulence of the econiche-associated, 3-oxo-dodecanoyl-L-homoserine (3OC_12_HSL)-producible *Pseudomonas aeruginosa* via farnesol-mediated signaling [16].

Unlike conventional antibiotics that either kill pathogens or directly inhibit growth with selective pressure consequently leading to the rise of resistant strains [17], [18], these chemical signaling molecules released from eukaryotes always diminish normal coordination of virulence gene expression in the associated prokaryotic pathogens without disturbance of their fundamental growth and survival. Such interkingdom cell-to-cell signaling molecules are thus considered a new-type of next-generational antibiotic against bacterial pathogens in medical and agricultural fields [19]–[21].

*Trichoderma*, an imperfect fungus, is a representative saprophyte that is highly interactive in root, soil and foliar environments. It has been developed into diverse commercial formulations, in particular, *Trichoderma* propagule-derived biopesticides have been successfully applied in field trials to control pathogens [22], [23]. Besides, owing to its antibacterial activity-guided bioassays, a wide array of *Trichoderma*-derived secondary metabolites, such as diketopiperazines [24], peptaibols, polyketides, terpenoids and pyrones [25] were isolated and identified. However, *Trichoderma*-derived secondary metabolites recognized as cell-to-cell signaling molecules have remained largely unknown [23], [26]. It is also unclear whether such *Trichoderma*-derived chemical substances regulate the physiological behavior of associating bacteria via interkingdom cell-to-cell signaling.

Among the relationships uncovered between *T. virens* PS1-7 and *B. plantarii*, it was found that *T. virens* PS1-7 repressed tropolone production of *B. plantarii*. During the search for the principle compound derived from *T. virens* PS1-7 that represses tropolone production, a non-antibacterial carotane-class sesquiterpene diol was isolated and characterized as a cell-to-cell signaling molecule produced by *T. virens* PS1-7. To investigate the mode of action of this sesquiterpene diol on *B. plantarii*, we examined the physiological and morphological changes of *B. plantarii* following exposure to tropolone or the exogenous sesquiterpene diol. In this paper, we describe an inhibitory effect of the sesquiterpene diol produced by *T. virens* PS1-7 on the virulence of blight-causative *B. plantarii* in association with its biofilm formation.

## Results

### Regression of Tropolone Production in *B. Plantarii* by *T. Virens* PS1-7 in a Coculture System

**Figure 1:**
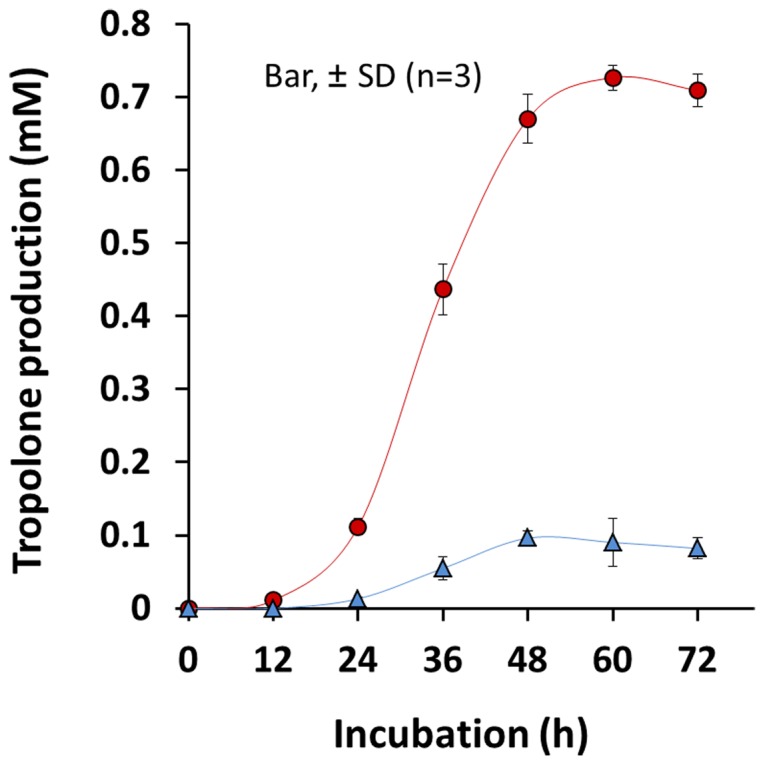
Tropolone production by *B. plantarii* in the coculture system. Tropolone production was quantified in the mono-culture of *B. plantarii* (red circle), and in the co-culture system of *B. plantarii* and *T. virens* PS1-7 (blue triangle). Values are means ± SD (shown as error bars) (n = 3).

In the monoculture, tropolone production of *B. plantarii* was maintained from 12 h to 72 h and reached a maximum of 0.73 mM at 60 h. In the coculture with *T. virens* PS1-7, tropolone production was drastically repressed throughout the time course and the maximum level of tropolone was reduced to approximately 1/7 of the monoculture (Fig. 1). Active principles repressing tropolone production by *B. plantarii* were examined among the fractionated samples of the secondary metabolites extracted from the culture fluid of *T. virens* PS1-7 in a tropolone production-repression activity assay.

### Carot-4-en-9,10-diol Attenuating Virulence of *B. Plantarii* on Rice Seedlings

**Figure 2:**
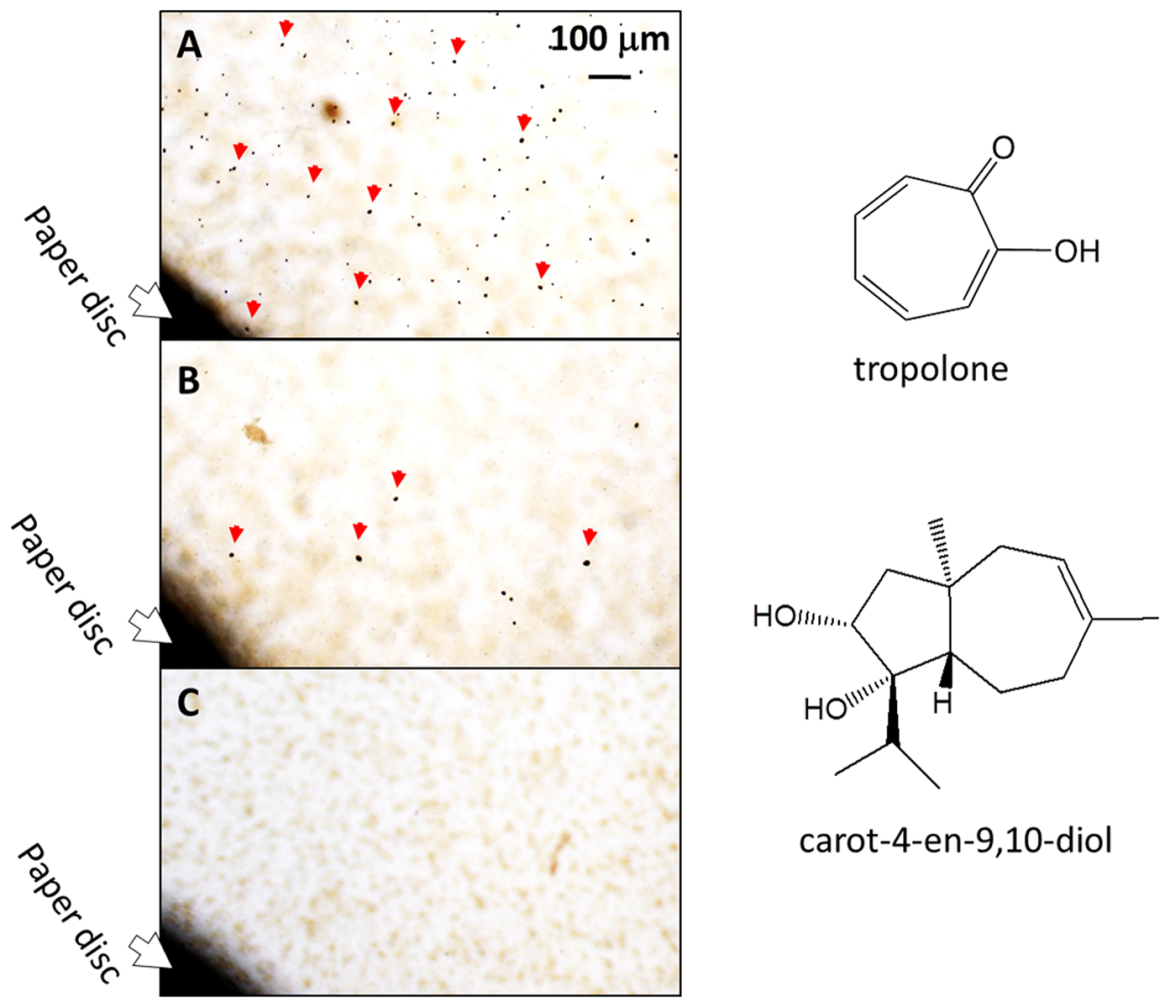
Active principle from *T. virens* PS1-7 for repression of tropolone production by *B. plantarii*. Tropolone production was semi-quantified by the density of dark crystallines formed by chelation of *B. plantarii*-produced tropolone with iron supplemented to the medium at 0.1 mM. Repression of tropolone production was observed in the area around the paper disc charged with solvent (A), the area around the paper disc charged with fraction 2 equivalent to 3 ml culture fluid (35 µg disc^−1^) (B), and with fraction 3 equivalent to 3 ml culture fluid (55 µg disc^−1^) (C). Red arrow indicates the typical tropolone-iron crystallines. Major component in the fractions 2 and 3 were identical with carot-4-en-9,10-diol. Its chemical structure including the relative configuration was shown in this figure.

**Figure 3:**
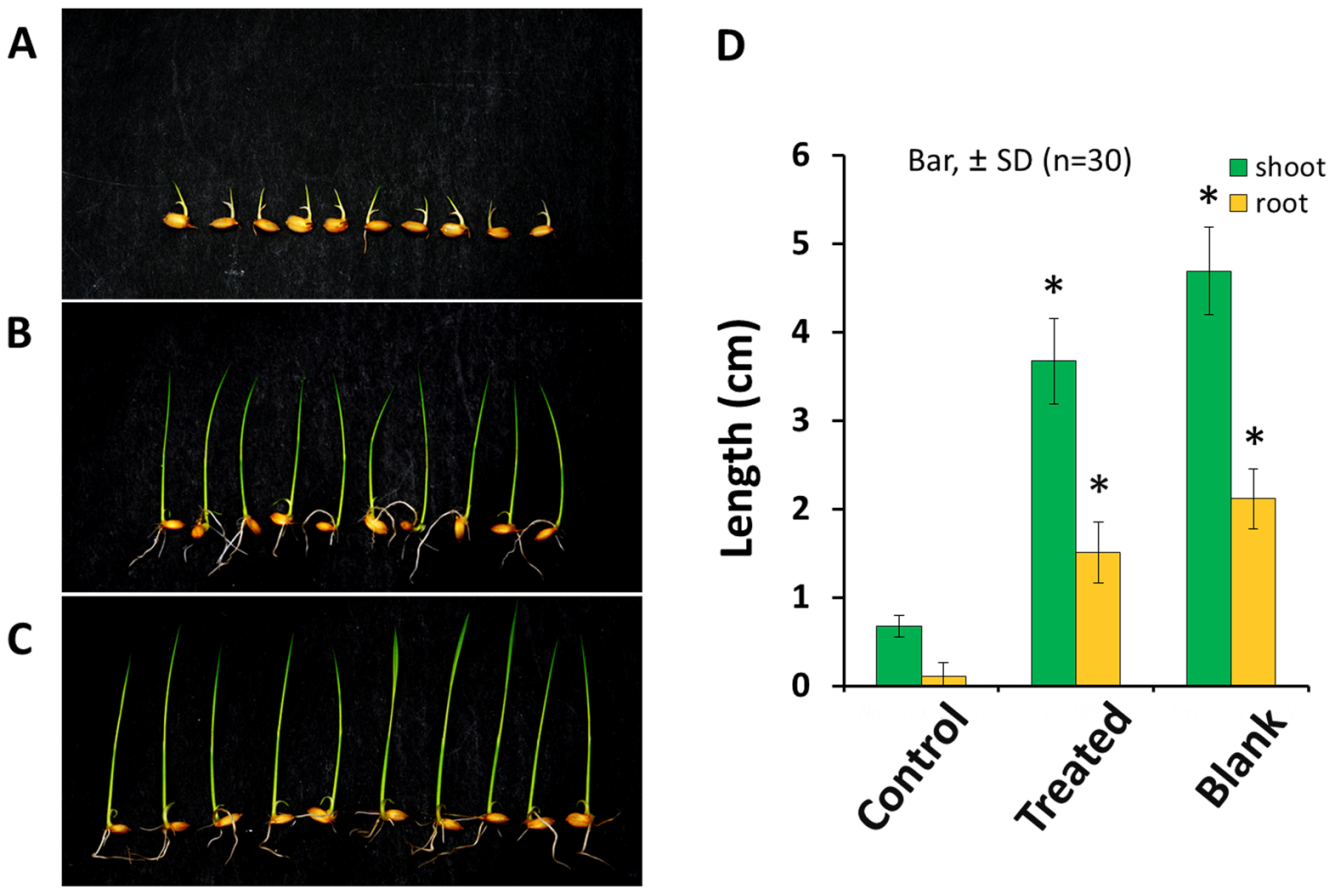
Virulence-attenuation effect of carot-4-en-9,10-diol on the growth of the rice seedlings inoculated with *B. plantarii.* Typical root and shoot growth performance among rice seedlings inoculated with *B. plantarii* (control, A), *B. plantarii*-inoculated rice seedlings that were also treated with 20 µM carot-4-en-9,10-diol at the same time (treated, B), inoculated rice seedlings without any inoculation of *B. plantarii* (blank, C). Virulence of *B. plantarii* recorded as the shoot and the root growth inhibition as indexes of the symptom was attenuated with statistical significance (D, right panel). Values are means ± SD (shown as error bar) (n = 30). *P<0.01 by Student’s-*t* test.

Fractions 2 and 3 drastically repressed tropolone production by *B. plantarii* at 35 µg disc^−1^ and 55 µg disc^−1^ (equivalent to 3 ml of the culture fluid), respectively, from this, the active principle was isolated (structurally identical to carot-4-en-9,10-diol) (Fig. 2). This sesquiterpene diol was an autoregulatory signal of *T. virens* PS1-7 responsive to tropolone [3]. In the following virulence assay, the rice seedlings (Koshihikari) infested with *B. plantarii* exhibited inhibition of growth in the root and shoot, while the rice seedlings infested with *B. plantarii* that had been treated with carot-4-en-9,10-diol exhibited similar growth performance to the control rice seedlings that had not been inoculated with *B. plantarii* (Fig. 3). This indicates that attenuation of *B. plantarii* virulence in rice seedlings is highly associated with repression of tropolone production mediated by carot-4-en-9,10-diol. To investigate the mode of action of carot-4-en-9,10-diol, we further analyzed the effects of the sesquiterpene diol on tropolone production, cell growth and cell morphology of *B. plantarii*.

### Repression of Tropolone Production and Induction of Cell Aggregation Without Cell Growth Inhibition in *B. Plantarii* Exposed to carot-4-en-9,10-diol

**Figure 4:**
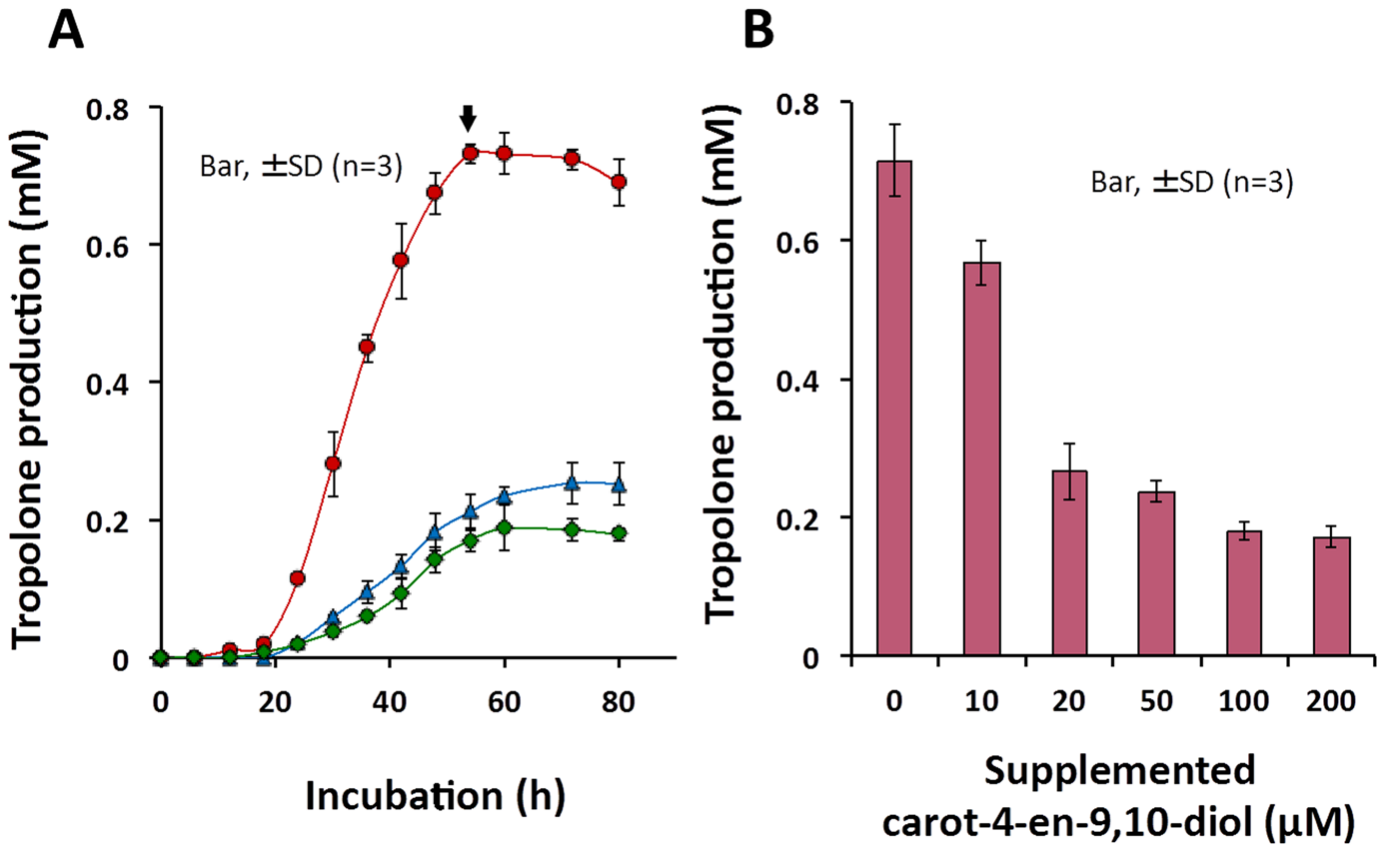
Effect of carot-4-en-9,10-diol on *B. plantarii* tropolone production. (A) Tropolone production was quantified from cultures of *B. plantarii* in PDB containing carot-4-en-9,10-diol at 20 µM (blue triangle), 200 µM (green diamond), and in the PDB without carot-4-en-9,10-diol (red circle). (B) Tropolone was analyzed quantitatively at 72 h for culture medium inoculated with *B. plantarii* containing carot-4-en-9,10-diol at zero, 10, 20, 50, 100 and 200 µM. Values are means ± SD (shown as error bar) (n = 3).

**Figure 5:**
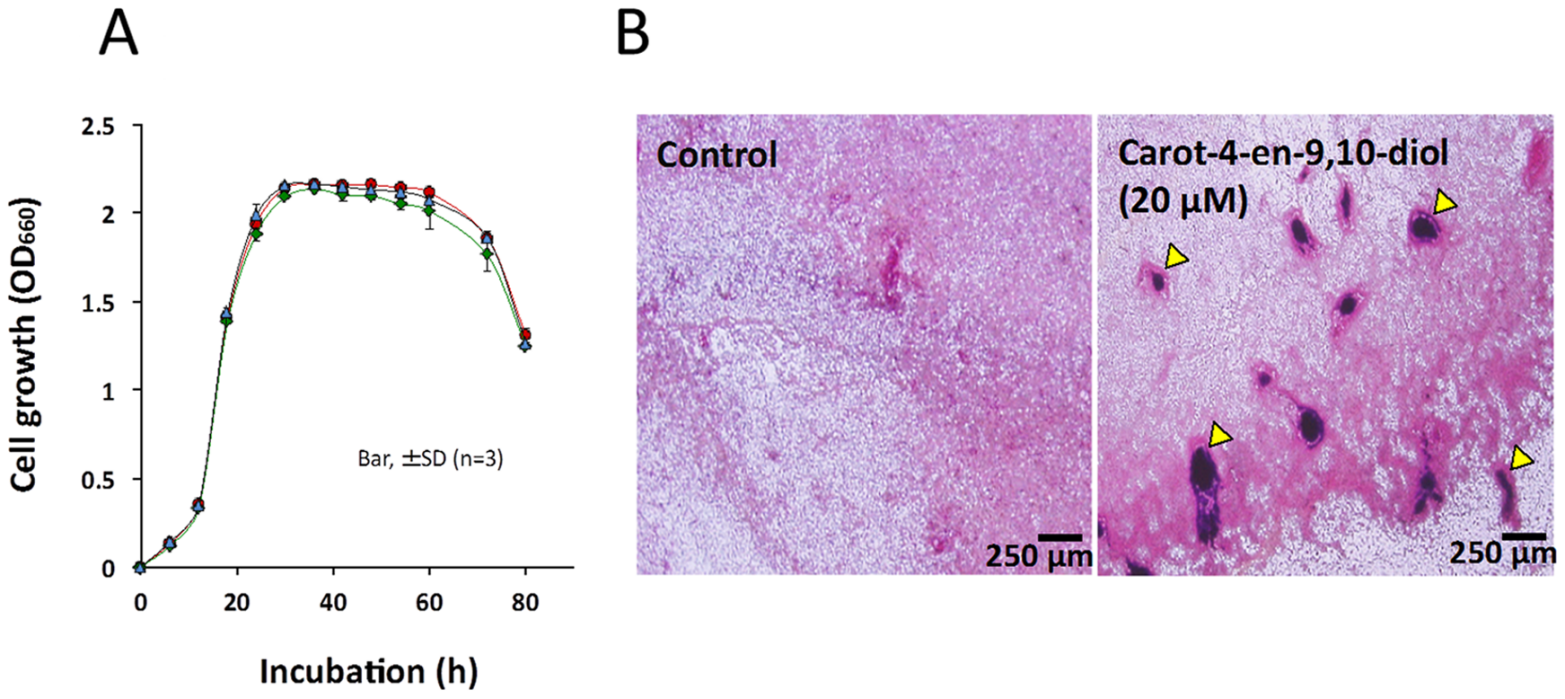
Effect of carot-4-en-9,10-diol on *B. plantarii* cell growth and morphology. (A) Cell growth was quantified from *B. plantarii* PDB cultures containing carot-4-en-9,10-diol at 20 µM (blue triangle), 200 µM (green diamond), and in PDB without carot-4-en-9,10-diol (red circle). Values are means ± SD (shown as error bars) (n = 3). (B) Cell morphology was observed at 30 h for culture medium inoculated with *B. plantarii* containing no carot-4-en-9,10-diol (left panel, control) and 20 µM (right panel). Yellow arrowheads indicate typical cell aggregation.

Normally, tropolone production in *B. plantarii* starts to rise during the exponential phase after an 18-h incubation and reaches a maximum level during the stationary phase at 54 h, at which point it becomes relatively stable (Fig. 4A, arrow). Upon exposure to carot-4-en-9,10-diol at either 20 µM or 200 µM, repression of tropolone production in *B. plantarii* was observed from the exponential to the stationary phase (Fig. 4A). In addition, the repression of tropolone production by carot-4-en-9,10-diol was dose-dependent in the range between 10 and 200 µM (Fig. 4B). Unlike tropolone production, cell growth of *B. plantarii* was not affected by carot-4-en-9,10-diol even at 200 µM (Fig. 5A). However, microscopic observation of *B. plantarii* cellular morphology showed that cell aggregation, which is the initial stage of bacterial biofilm formation, was induced at the early stationary phase by 20 µM or higher concentrations of supplemented carot-4-en-9,10-diol (Fig. 5B).

### *B. Plantarii* Biofilm Formation Promoted by Tropolone or carot-4-en-9,10-diol

**Figure 6:**
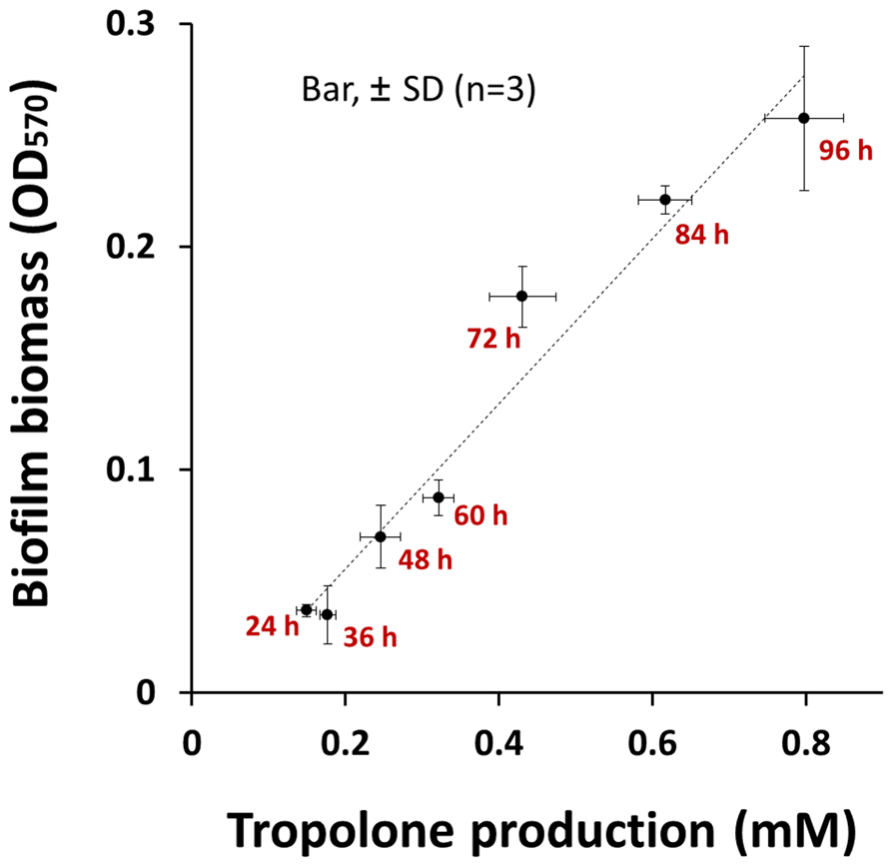
Relationship between biofilm formation and tropolone production in *B. plantarii*. Correlation analysis of biofilm biomass and endogenous tropolone produced by *B. plantarii* incubated for 24, 36, 48, 72, 84 and 96 h in the static culture system. The endogenous tropolone production showed a positive and linear correlation with the biofilm formation evaluated by crystal violet staining method with the correlation coefficient of *r* ^2^ = 0.96 (n = 18). Both tropolone production and biofilm formation increased along with longer incubation time up to 96 h. Values shown by the plots are means ± SD (n = 3). Vertical and horizontal error bars on each plot were for the biofilm biomass (OD_570_) and the tropolone production (mM) respectively.

A positive correlation between biofilm biomass and endogenous tropolone production by *B. plantarii* gave the following linear equation: y  = 0.37× - 0.018 (*r^2^* = 0.96) (Fig. 6), suggesting that extracellular accumulation of endogenous tropolone is required for autoinducing *B. plantarii* biofilm formation. Besides, iron (FeCl_3_), which is known to reduce endogenous tropolone by forming an iron-tropolone complex, reduced biofilm formation dose-dependently at concentrations less than 500 µM (Figure A in File S1). Moreover, endogenous tropolone-regulated biofilm formation was further promoted by supplementation of exogenous tropolone dose-dependently at concentrations less than 200 µM (Figure B in File S1). These results supported the hypothesis that *B. plantarii* biofilm formation was regulated by tropolone, a biofilm formation-autoinducing signal.

When *B. plantarii* was exposed to carot-4-en-9,10-diol, accumulation of endogenous tropolone in the culture fluid was drastically reduced (Fig. 4B), but biofilm formation was instead promoted rather than being inhibited. This unique response by *B. plantarii* to carot-4-en-9,10-diol during biofilm formation seemed to be similar to that promoted by exogenous tropolone (Figures A–D in File S2). In addition, biofilm formation was also induced by co-treatment with exogenous tropolone and carot-4-en-9,10-diol (Figures C–D in File S2).

### Morphological and Physiological Differences between the *B. Plantarii* Biofilms Induced by Exogenous Tropolone and carot-4-en-9,10-diol

**Figure 7:**
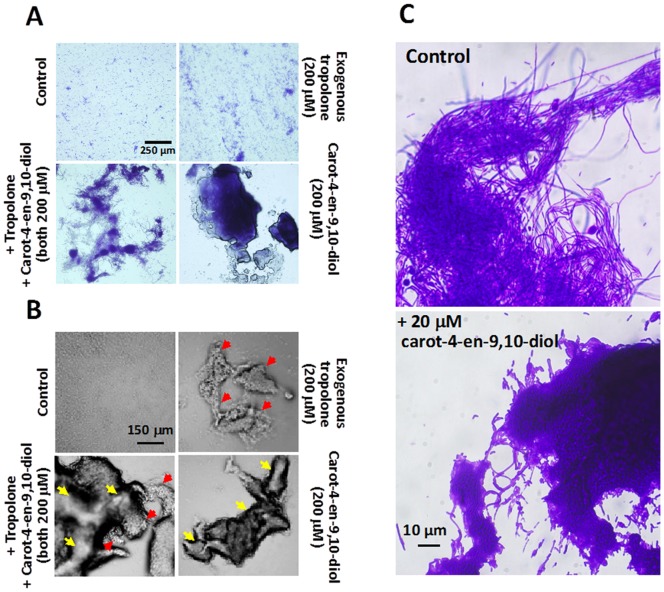
Morphological and physiological characteristics of biofilms formed by *B. plantarii* exposed to tropolone or carot-4-en-9,10-diol. (A) General state of *B. plantarii* biofilm formation after a 48 h incubation in PDB containing 200 µM exogenous tropolone only (top, right panel), 200 µM exogenous tropolone plus 200 µM carot-4-en-9,10-diol (bottom, left panel), 200 µM carot-4-en-9,10-diol (bottom, right panel), and in PDB containing neither exogenous tropolone nor carot-4-en-9,10-diol as control (top, left panel). The microscopic observation was done with a 10× objective lens, after the cells were stained by a crystal violet. (B) Three dimensional structures of representative cell aggregates in each treatment after 48 h incubation were also observed under a phase contrast mode, with the same relative positions of each panel with that of A. Red arrows indicate the biofilm induced by tropolone, while yellow arrows indicates the biofilm induced by carot-4-en-9,10-diol. (C) Comparison of matrix of the biofilm formed by *B. plantarii* incubated for 96 h with endogenous tropolone (top panel, control) or 20 µM carot-4-en-9,10-diol (bottom panel). Black arrow indicates the typical fibrous matrix.

At the early stage, diverse biofilms formed by *B. plantarii* in response to tropolone or carot-4-en-9,10-diol mainly exhibited a general state of cell aggregation with the development of three dimensional structures. Under exposure to endogenous tropolone only, *B. plantarii* mostly formed small dispersive cell aggregates on the shallow-surface of the plate (controls in Fig. 7). With supplementation of exogenous tropolone (e.g. 200 µM), *B. plantarii* formed a similar biofilm, along with loose and fluffy floccule-like large cell aggregates (Fig. 7A and B).

Unlike the biofilms induced by endogenous/exogenous tropolone as described above, *B. plantarii* exposed to carot-4-en-9,10-diol formed few but much larger cell aggregates made of thick tight clumps. With supplementation of exogenous tropolone (200 µM) together with carot-4-en-9,10-diol (200 µM), *B. plantarii* also formed few but large cell aggregates that comprised a mixture of clumps and a portion of floccules. Thus, the *B. plantarii* biofilm induced by carot-4-en-9,10-diol at the early stage, is morphologically distinguishable from that induced by tropolone (Fig. 7A and B).

**Figure 8:**
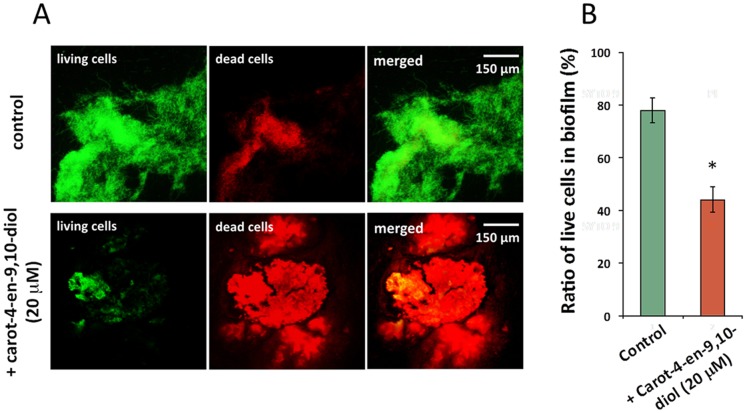
Cell viability assay for the biofilm induced by tropolone or carot-4-en-9,10-diol. Comparison of cell viability of the biofilms formed by *B. plantarii* that was incubated for 96 h in response to endogenous tropolone (control) or supplementation of 10 µM carot-4-en-9,10-diol was done by observation of fluorescently-labelled biofilms (A, left panels) and quantification of living/dead cells (B, right columns). In panels in A, cells showing green fluorescence are living cells, while red are dead. Values are means ± SD (shown as error bars) (n = 3). *P<0.01 by Student’s-*t* test.

In addition, some unique fibrous structures of *B. plantarii* biofilm were induced by endogenous or exogenous tropolone and were observed under a microscope. In contrast, these fiber-like cell structures were not observed in the biofilm induced by carot-4-en-9,10-diol (Fig. 7C). Particularly, at the late stage of biofilm formation, the biofilm mediated by endogenous tropolone (750 µM) showed an integrated fibrous-matrix (Fig. 7C, top panel), whereas the biofilm induced by 20 µM carot-4-en-9,10-diol was completely missing fibrous structure (Fig. 7C, bottom panel). Moreover, compared with the relatively high viability of the tropolone-mediated biofilm (Fig. 8A, top panels), the carot-4-en-9,10-diol-mediated biofilm showed remarkably low viability (Fig. 8A, bottom panels). Quantitative analysis using the ratio of live/dead cells showed 44% cell viability in the carot-4-en-9,10-diol-mediated biofilm in contrast to 78% cell viability in the biofilm mediated by tropolone (Fig. 8B). These results indicated that the biofilm induced by carot-4-en-9,10-diol was a non-functional biofilm physiologically different from the virulent-type biofilm mediated by tropolone.

### Inhibition of *Plai* Gene Expression in *B. Plantarii* Induced by Carot-4-en-9,10-diol and Quorum Sensing Inhibitors

**Figure 9:**
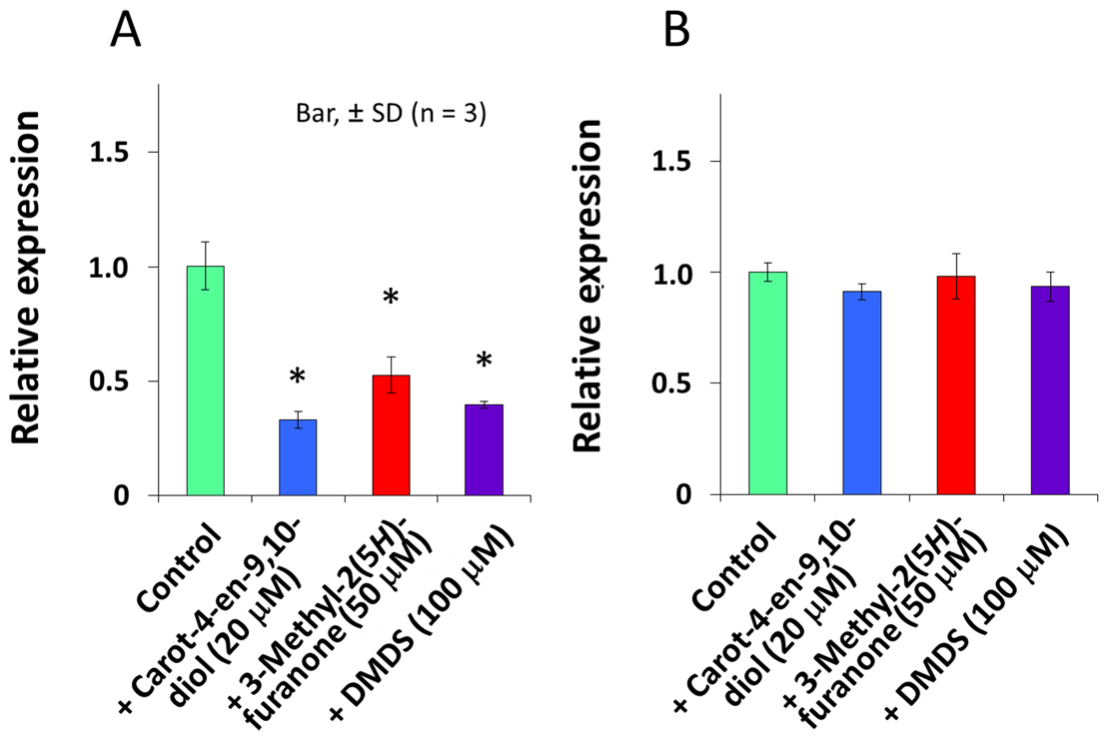
Quantitative real time PCR analysis of the effects of carot-4-en-9,10-diol and quorum sensing inhibitors on *plaI* (A) and *plaR* (B) gene expression in *B. plantarii*. *B. plantarii* was incubated in PDB containing 20 µM carot-4-en-9,10-diol, 50 µM 3*-*methyl*-*2(5*H*)*-*furanone, or 100 µM DMDS. Control as PDB containing solvent only. Values are means ± SD (shown as error bars) (n = 3). *P<0.01, **P<0.001 by Student’s-*t* test.

Considering the crucial role of the AHL-QS system in regulation of the pathogenicity of *B. plantarii* [27], we analyzed expression of the AHL-QS system associated genes *plaI* and *plaR* in *B. plantarii* using qRT-PCR. Two quorum sensing inhibitors (QSIs) against the bacterial AHL-QS system [28], [29], 3*-*methyl*-*2(5*H*)*-*furanone (TCI, Tokyo, Japan) and dimethyl disulfide (DMDS) (TCI, Tokyo, Japan), significantly inhibited the *t*ranscription level of *plaI* in *B. plantarii* at 50 µM and 100 µM doses, respectively (Fig. 9A). However, neither inhibitor affected *plaR* expression (Fig. 9B). Similarly, under exposure to carot-4-en-9,10-diol (20**µM), transcription levels of *plaI* were decreased nearly 70% in *B. plantarii* compared to the control (Fig. 9A), while *plaR* transcription levels were almost equivalent to the control (Fig. 9B). These results suggested that QQ [30] mediated by carot-4-en-9,10-diol was the main mode of action by which carot-4-en-9,10-diol repressed tropolone production.

## Discussion

Carot-4-en-9,10-diol, which was originally isolated from a strain of *T. virens* (*Gliocladium virens* IFO9166) [31], was recently found to be a conidiation-autoinducer in *T. virens* [3]. Carot-4-en-9,10-diol was produced by *T. virens* PS1-7 in response not only to chemical stress from catechol, tropolone and other iron chelators [3], but also to the coculture system with *B. plantarii*. Without any chemical stress, this carotane-type sesquiterpene accumulated significantly in the *T. virens* PS1-7 culture fluid at concentrations that ranged between 20 to 30 µM [3] and appeared to be responsible for the repression of tropolone production by *B. plantarii* (Fig. 4). During the bioassay-guided chase for tropolone production-repressing active substances from *T. virens* PS1-7, carot-4-en-9,10-diol was isolated and eventually characterized as the virulence-attenuating substance (Fig. 3). It is thus proposed that a new biological role for the carot-4-en-9,10-diol produced by *T. virens* PS1-7 is as an interkingdom cell-to-cell signaling molecule that regulates virulence of *B. plantarii*. Topically, the mode of action of carot-4-en-9,10-diol in the repression of tropolone production by *B. plantarii* as well as cellular responses of *B. plantarii* to carot-4-en-9,10-diol was investigated.

Production of a virulence factor in many plant-associated gram-negative bacteria determines their phytopathogenicity to host plants and is positively regulated by the QS system [32]–[35], e.g. toxoflavin production by *Burkholderia glumae* under control of AHL-QS system [36]. Previously, using a gene-knock-out-technique in combination with a bioassay, Solis *et al.* revealed that AHL-QS system positively regulated the pathogenicity of *B. plantarii* to rice seedlings [27]. However, it was not clear whether the AHL-QS system positively regulated tropolone production, which determines the pathogenicity of *B. plantarii* to rice seedlings [2]. Our direct analysis of the relationship between tropolone production and cell density showed that tropolone production by *B. plantarii* was cell density-dependent (Figure A in File S3), thus demonstrating the AHL-QS-controlled tropolone production. Furthermore, tropolone production in *B. plantarii* was significantly repressed by carot-4-en-9,10-diol (Fig. 4B), irrespective of its cell growth. In addition, both commercially available QSIs *3-*methyl-2(5*H*)-furanone and DMDS [28], [29] significantly repressed tropolone production in *B. plantarii* with the minimum dosage respectively at 100 µM and 200 µM (Figures B–C in File S3). Neither of these doses significantly interfered with bacterial cell growth (Figures B–C in File S3). Together, these results indicate that carot-4-en-9,10-diol is likely to target the AHL-QS system in *B. plantarii* as do other known QSIs.

Quantitative RT-PCR analysis showed that the QQ mediated by carot-4-en-9,10-diol only led to inhibition of expression of the gene *plaI* in the *B. plantarii* AHL-QS system (Fig. 9A), and not the gene *plaR* (Fig. 9B). In *B. glumae* presence of the AHL-QS system, which is highly homologous with *B. plantarii*, *tofR*-encoded AHL receptor TofR formed an AHL-TofR complex activator, which activated expression of downstream genes and positively regulate the expression of the AHL synthase gene *tofI* [36]. This finding together with our current analysis indicates that in *B. plantarii*, expression of *plaR* was not inhibited by carot-4-en-9,10-diol (Fig. 9B) and thus production of the *plaR-*encoded AHLs receptor (PlaR) was also not affected. However, carot-4-en-9,10-diol was likely to be a chemical signal mimic of AHL that competitively binds to PlaR leading to disruption of normal formation of the AHL-PlaR complex [6], thus blocking normal expression of *plaI* (Fig. 9A). It further caused repressed production of the AHL synthase PlaI and thus AHL, and consequently amplified the disruption of the AHL-QS signaling circuit by caro-4-en-9,10-diol [36], [37].

*B. plantarii* biofilm formation was triggered by endogenous tropolone (Fig. 6) that is another new discovery. Indeed, self-produced endogenous secondary metabolites other than AHLs are often found to be an autoinducing signal molecule for biofilm formation in many other bacteria [38]–[40]. For example, a tropolone derivative, tropodithietic acid (TDA) is characterized as an autoinducer-type signaling molecule of *Silicibacter* sp. TM1040 owing its inducible effect on TDA production and biofilm formation [41], [42]. Similarly, *B. plantarii* biofilm formation demonstrated a natural development process in response to the autoinducer signaling molecule tropolone [43], which was significantly accelerated with supplementation of exogenous tropolone (Figure B in File S1). This indicates that exogenous tropolone may further induce production of endogenous tropolone and synchronously promote biofilm formation.

*B. plantarii* showed distinguishable morphological responses to endogenous/exogenous tropolone and exogenous carot-4-en-9,10-diol in different states of biofilm formation (Fig. 7A and B). As an AHL signal mimic, carot-4-en-9,10-diol repressed AHL-QS-controlled tropolone production of *B. plantarii* (Fig. 4B), leading to a reduction in tropolone-mediated biofilm formation (Fig. 6). Conversely, *B. plantarii* is likely to perceive carot-4-en-9,10-diol as an exogenous chemical stimuli different from its endogenous chemical molecules, and in response, assemble more cells into larger aggregates as was observed during the different states of biofilm formation [44].

This response of *B. plantarii* to carot-4-en-9,10-diol is similar to that shown in previous findings demonstrating that some bacteria promote biofilm formation when they are exposed to sub-inhibitory concentrations of exogenous antibiotics that fail to inhibit their cell growth, despite morphological differences [36]. The mechanisms by which these exogenous chemical stimuli as well as self-produced autoinducer signaling molecules, regulate bacterial biofilm formation is not well understood [36]. However, the different states of *B. plantarii* biofilm formation in response to tropolone and carot-4-en-9,10-diol (Fig. 7A and B) suggest that the complex intracellular signaling pathways are involved in positive regulation of biofilm formation diversely modulated by the low molecular signal compounds and their mimics [45]–[47].

Along with *B. plantarii* biofilm formation due to exposure to carot-4-en-9,10-diol, defective matrix (Fig. 7C) and decreased cell viability (Fig. 8) were uniquely observed in the late stage. Unlike the biofilm induced by endogenous tropolone which is exclusively made up of fibrous matrix, the biofilm induced by carot-4-en-9,10-diol comprised a large number of nonviable cells and had defects in matrix integrity, suggestive of a pseudo-biofilm [48]. Since these physiological and morphological defects of the pseudo-biofilm were restored by supplementation of exogenous tropolone (data not shown), the tropolone-deficient environment seems to be the main factor causing abnormal development of biofilm [49]. Taken together, these results indicate that tropolone not only functions as a virulence factor and an autoinducer that triggers biofilm formation, it also acts as an antioxidant redox signal which maintains normal biofilm development in the lifecycle of *B. plantarii* [49], [50].

In conclusion, tropolone produced by *B. plantarii* is an autoinducer that mediates biofilm formation. Moreover, carot-4-en-9,10-diol, which is released by *T. virens* PS1-7, functions as a cell-to-cell signal mimic towards the rice seedling blight-causative agent, *B. plantarii* to repress tropolone production and induce abnormal biofilm, both of which led to attenuation of virulence. Collectively, the findings presented in this study demonstrate that future insight into fungus-bacterium interactions may provide novel ways for modulation of bacterial virulence. These highlights further serve as a basis for development of sesquiterpene-type chemical regulators that attenuate virulence of *B. plantarii*.

## Materials and Methods

### Chemicals and Analytical Instruments

The main chemicals and analytical instruments used in this study are as follows: authentic tropolone (Wako, Osaka, Japan), 3*-*methyl*-*2(5*H*)*-*furanone (TCI, Tokyo, Japan), and dimethyl disulfide (TCI, Tokyo, Japan)*;* Waters 600 HPLC (Waters, MA, USA) installed with an L-column2 ODS column (250 mm by 4.6 mm; i.d. 5 µm), MS spectrometers JEOL JMS-T100GCV and JMS-SX-102 (JEOL, Tokyo, Japan), NMR spectrometer JEOL JNM-EX270 (JEOL, Tokyo, Japan), ABI Prism 310 genetic analyzer (Applied Biosystems, CA, USA), Takara TP800 thermal cycler real time dice (Takara, Tokyo, Japan).

### Microbial Strains, Growth Media and Culture Conditions

*T. virens* PS1-7, a tropolone-resistant fungus, was isolated from rice rhizosphere previously [3]. *B. plantarii* was provided by Professor Yuichi Takikawa (Faculty of Agriculture, Shizuoka University) via Kumiai Chemical Industry Co. *T. virens* PS1-7 and *B. plantarii* were routinely grown at 25°C in the dark in potato dextrose broth (1× PDB, pH 6.2) at either statically or at 110 rpm. Alternatively, cultures were grown on a potato dextrose agar (PDA) plate that was solidified with 1.5% agar (Wako, Osaka, Japan).

### Coculture System for Tropolone Production by B. Plantarii Grown with T. Virens PS1-7

Tropolone production by *B. plantarii* cocultured with *T. virens* PS1-7 in PDB was analyzed according to the time course for incubation. For the coculture system, 50 µl of the cell suspension of *B. plantarii* (10^5^ CFU ml^−1^) and conidia suspensions of *T. virens* PS1-7 (10^5^ conidia ml^−1^) in sterile phosphate-buffered saline (PBS, pH 7.4) were both inoculated into 5 ml of PDB in a sterilized 18-cm test tube at 25°C in the dark. Monocultured *B. plantarii* was used for the control. The resulting cultures were shaken for 0, 12, 24, 48, 60 and 72 h, before being subjected to solid-phase extraction (SPE) and HPLC for quantification of tropolone as described in the previous report [3]. Each sampling was done in triplicate.

### Isolation and Identification of the Tropolone Production-repression Principle from *T. Virens* PS1-7

To obtain secondary metabolites of *T. virens* PS1-7, 10^6^ conidia of *T. virens* PS1-7 was inoculated in 3 liters of PDB and shake-cultured for 72 h at 25°C in the dark. The resulting culture fluid filtered through no. 101 filter paper (Advantec, Tokyo, Japan) was extracted exhaustively with ethyl acetate (EtOAc) (500 ml × 6). The organic layer was combined, dried over anhydrous Na_2_SO_4_, and then concentrated. Crude extracts yielded (715 mg) were dissolved in *n*-hexane-EtOAc (v/v, 95∶5) and subsequently subjected to chromatography in a silica gel column (50 g, GF_60_ 35 to 70 mesh, Merck, Darmstadt, Germany) by stepwise elution with 5% to 100% EtOAc in *n*-hexane.

Six main fractions were subjected to an agar diffusion assay on the *B. plantarii*-impregnated PDA containing 0.1 mM FeCl_3_ for chasing the tropolone production-repressing principles [51]. Each fraction was dissolved in 5 ml of EtOAc, and then diluted sequentially by 10 fold. After passage through a 0.2 µm sterilizing filter syringe, 50 µl of each fraction was applied to a 8-mm-diamete paper disc (thick type, Advantec). As the control, paper discs were only charged with the same volume solvent. After a 3-d incubation, formation of visible and ovate complex iron-tropolone crystalline precipitates (20∼30 µm) around the paper discs was examined under a light microscope (Olympus ix70, Tokyo, Japan) in order to assess the repression of tropolone production. Consequently, fraction 2 (35 mg, eluted with 30% EtOAc) and fraction 3 (55 mg, eluted with 40% EtOAc) had the ability to repress tropolone production.

Fractions 2 and 3 that uniquely repressed tropolone production in *B. plantarii* were subjected to thin layer chromatography (TLC) (Kieselgel 60 GF_254,_ 0.25 mm, Merck, Darmstadt, Germany) developed in an EtOAc and hexane solution (v/v 3∶2). A purple spot (*Rf* value, 0.76) formed after spraying with a vanillin-sulfuric acid reagent followed by heating. This was characterized as the major component and obtained as colorless needles (37.9 mg) with preparative TLC. Spectroscopic data obtained by FD-MS, FD-HR-MS (JEOL JMS-T100GCV) and EI-MS (JMS-SX-102) were as follows: FD-MS 238.2 (100, [M]^+^), FD-HR-MS [M]^+^ founded at *m/z* 238.1936 (C_15_H_26_O_2_, calcd. 238.1932); EI-MS: at *m/z* (rel. int. %), 238 (13, [M]^+^), 220 (11, [M-H_2_O]^+^), 202 (12, [M-2H_2_O]^+^), 195 (98, [M-Me_2_CH]^+^), 177 (100), 159 (69), 123 (40), 107 (42), 93 (42), and 43 (88). These chromatographic and spectroscopic data showed that the active compound obtained by the bioassay-guided isolation was identical as carot-4-en-9,10-diol, which had previously been reported as an autoregulatory signal molecule of *T. virens* PS1-7 (Files S4 and S5) [3]. In comparisons of ^1^H and ^13^C-NMR (JEOL JNM-EX270) chemical shift values of the active principle with those reported in other strains of *T. virens* [31], [52], [53], chemical structure of carot-4-en-9,10-diol was finally confirmed.

### Virulence-attenuation Assay for carot-4-en-9,10-diol

Healthy rice seeds (*Oryza sativa* cv. Koshihikari) were surface-sterilized according to a procedure reported previously [3] and inoculated with *B. plantarii* by soaking them in a petri dish containing 10 ml of bacterial cell suspension (10^3^ CFU ml^−1^). Surface-sterilized rice seeds not infested with *B. plantarii* were also prepared. Subsequent transplantation and incubation were performed according to our previous report [3], *B. plantarii*-infested rice seeds were transplanted in a seed bed supplemented with caro-4-en-9,10-diol in the range from 10 to 200 µM (treated). Seed beds without carot-4-en-9,10-diol, were also transplanted with *B. plantarii*-infested rice seeds (control) or rice seeds without inoculation of *B. plantarii* (blank). Growth performance of rice seedlings recorded by the length of the shoot and the root was used to assess virulence of *B. plantarii*.

### Tropolone Production, Cell Growth and Morphology of *B. Plantarii* Exposed to Carot-4-en-9,10-diol

Carot-4-en-9,10-diol dissolved in dimethyl sulfoxide (DMSO) was diluted into a series of consisting of stock solutions at concentrations of 10, 20, 50, 100 and 200 mM. A five-µl portion of each solution was added to a 5 ml PDB in the sterilized 18-cm test tube. PDB with DMSO only was used as the control. These PDB media containing 0, 20 and 200 µM of carot-4-en-9,10-diol along with control were each inoculated with *B. plantarii* (10^3^ CFU ml^−1^) and shake-cultured for time course experiments lasting 0, 6, 12, 18, 24, 30, 36, 42, 48, 54, 60, 72 and 80 h in order to monitor tropolone production and cell growth. The tropolone produced was quantified as described previously [3] and cell growth was monitored by optical density of the culture (at 660 nm). To examine the effect of carot-4-en-9,10-diol on the tropolone production by *B. plantarii*, cultures that had been incubated for 72 h were also performed in PDB containing 0, 10, 20, 50, 100 and 200 µM of this sesquiterpene diol and were taken and subjected to quantification of tropolone production by HPLC. After a 30-h incubation of *B. plantarii* cells in the medium containing 20 µM carot-4-en-9,10-diol, the morphological characteristics of the cultured cells subjected to Gram staining was observed under the light microscope (Olympus ix70).

### Biofilm Formation assay for *B. Plantarii* Exposed to Exogenous Tropolone, Iron and Carot-4-en-9,10-diol

Authentic tropolone (Wako, Osaka, Japan) and carot-4-en-9,10-diol were each dissolved in DMSO as a 10 mM stock solution, while FeCl_3_ was dissolved in Milli-Q water to be a 10 mM stock solution. *B. plantarii* statically incubated in a plastic petri dish (35 × 10 mm, BD Falcon, NJ, USA) was used for the biofilm formation assay. According to the previous protocol [54], quantification of biofilm biomass was done using the crystal violet (CV) staining method, and microscopic observation was done using a Biorevo BZ-9000 (Keyence, Osaka, Japan) in normal objective mode for CV-stained biofilm and phase contrast objective mode for non CV-stained biofilm.

To investigate biofilm formation of *B. plantarii*, the static cultures incubated for 24, 36, 48, 60, 72, 84 and 96 h were respectively subjected to quantification of biofilm biomass along with quantitative analysis of tropolone produced in the cultures. To investigate the effect of tropolone on biofilm formation, biofilm biomass of *B. plantarii* grown under tropolone-enriched conditions by adding exogenous tropolone to either 100 or 200 µM was quantified at 24, 48, 72 and 96 h, respectively. In addition, biofilm biomass of *B. plantarii* grown under tropolone-quenched conditions in the presence of FeCl_3_ as 200 and 500 µM was also monitored at 24, 48, 72 and 96 h. Each control contained the same volume of DMSO or water.

To compare the effect of carot-4-en-9,10-diol and tropolone on *B. plantarii* biofilm formation, quantification of biofilm biomass was done in parallel with microscopic observation after a 48-h incubation in the three treatments using PDB containing 200 µM exogenous tropolone, 200 µM carot-4-en-9,10-diol, or 200 µM exogenous tropolone plus 200 µM carot-4-en-9,10-diol. PDB containing the same volume of DMSO was used for the control.

To observe the biofilm induced by carot-4-en-9,10-diol, *B. plantarii* was incubated for 96 h in PDB with or without 20 µM carot-4-en-9,10-diol. The planktonic cells were removed and 2 ml of PBS was added to detach the biofilm mechanically through pipetting [55]. For observation of matrix structure, the detached biofilms were washed with PBS several times, placed on a glass slide, fixed using a flame and then stained with 0.1% CV solution at room temperature for 1 min. The CV-stained biofilm was washed with water excessively and then observed with oil immersion under a light microscope at 100 × (Olympus ix70).

For observation of cell viability in the biofilm, the detached biofilms were subjected to fluorescence staining with Live/Dead *Bac*Light Bacteria Viability Kit L13152 (Molecular Probes, Eugene, OR, USA) according to the manufacturer’s instructions and the fluorescence-labelled biofilms were observed under the Biorevo BZ-9000. The living cells stained with SYTO 9 were detected as green fluorescence with a GFP-BP filter (Ex 470/40, Em 535/50), while the dead cells stained with propidium iodide were detected as red fluorescence with a TRICC filter (Ex 540/25, Em 605/55).

For accurate quantification of cell viability, the resulting biofilm was re-suspended in 1 ml of PBS mixed with 0.2 g sterilized zirconia beads (0.1 mm, YZB01, Yasuikikai, Osaka, Japan) and vortexed at 2500 rpm for 1 min to separate the cells from the matrices [56]. After standing for 5 min, cell suspensions in the up layer were collected and adjusted into the equivalent cell density (OD_660_ = 0.02) and relative fluorescence intensity was measured in a microplate reader (Tecan Rainbow Thermo, Männedorf, Switzerland) as per the manufacturer’s recommendations. Ratio of living-dead cells was calculated by dividing green fluorescence intensity (Ex 485, Em 530) with red fluorescence intensity (Ex 485, Em 630).

### Response of *B. Plantarii* to Cell Population Density in Association with Tropolone Production

The relationship between tropolone production of *B. plantarii* and cell population growth was investigated as follows: *B. plantarii* cells were collected from 24 h cultures incubated with shaking and harvested by centrifugation at 8,500 × *g* for 2 min. Cells were re-suspended with 10 ml of PBS in a 50 ml-Falcon tube at approximately 10^8^ CFU ml^−1^ (OD_660_ = 1.0), and serially diluted in 10 ml of PBS to 10^7^, 10^6^, 10^5^, 10^4^ and 10^3 ^CFU ml^−1^. After removal of PBS by centrifugation at 8,500 × *g* for 2 min, a 10 ml liquefied PDA kept at 40°C was poured into the tube with the precipitated cells, which was then rapidly mixed and poured onto a new petri dish.

After a 72-h incubation, the PDA that had been inoculated with *B. plantarii* was homogenized in 10 ml of PBS, and a 2 ml-portion of the resulting homogenates was centrifuged at 10,000 × *g* for 2 min after which 1.5 ml of the supernatant was subjected to quantification of tropolone production. A 50 µl-portion from the relevant homogenates were appropriately diluted in sterilized water, spread onto a PDA plate and incubated for 24-h, in order to count the colony forming units (CFU ml^−1^) for determination of cell populations.

### Effect of Quorum Sensing Inhibitors on Tropolone Production by *B. Plantarii*

To investigate effect of quorum sensing inhibitors (QSIs) on tropolone production in *B. plantarii,* two types of representative QSIs, furanone derivative and volatile organic compound (VOCs), 3-methyl*-*2(5*H*)*-*furanone and dimethyl disulfide (DMDS) [28], [29], [57] were selected and dissolved in DMSO at 50, 100 and 200 mM stock solutions. A 5 µl-portion from the stock solution was added to 5 ml of PDB to make 50, 100 and 200 µM final concentrations. PDB containing 5 µl DMSO was used as a control. *B. plantarii* (10^3^ CFU ml^−1^) inoculated in these PDB media was cultured with shaking for 24 h for determination of cell growth, while incubation for 72 h was used for the quantification of tropolone produced in the culture fluid.

### Quantitative RT-PCR for *plai-R* Gene Expression in *B. Plantarii* Exposed to Carot-4-en-9,10-diol and Quorum Sensing Inhibitors

*B. plantarii* cells cultured overnight with shaking were collected and subjected to extraction of genomic DNA using an Isoplant II kit (Nippon Gene, Toyama, Japan). Based on Align-BLAST (NCBI), the alignment of coding sequences (CDS) for LuxI family acyl-homoserine-lactone synthase and quorum sensing LuxR family sensor regulator in *B. plantarii* (strain ATCC 43733, accession no. AM086212) and *B. glumae* (strain BGR1, chromosome DNA accession no. NC012721) were obtained. Each primer pair for PCR-amplification of *plaI* and *plaR* in *B. plantarii* was then designed using Primer-BLAST (NCBI) as: *plaI* forward 5′-TCGTACCGTTATCGCGTGTT-3′ and *plaI* reverse 5′-GAACGT GACCCCGATCAACT-3′; *plaR* forward 5′-TCTTTCACCAGGTTTCCGCA-3′ and *plaR* reverse 5′-AGCTTGGCGAGGATGTTGTT-3′, respectively. The DNA sequences of PCR-amplicons of *plaI* and *plaR* were identified and respectively deposited in the DNA Data Bank of Japan (DDBJ) with accession no. AB787149 and AB787150. Using these sequences, specific-primers for quantitative RT-PCR (qRT-PCR) were accordingly designed using Primer-BLAST as: *plaI* RT-forward 5′-GGAAGACGAAAAATTCGAG-3′ and *plaI* RT-reverse 5′-TACACCGGTATCGTCG-3′; *plaR* RT-forward 5′-GAGATCAACAGCCTGAC-3′ and *plaR* RT-reverse 5′-AGCGAATGCGAGAGAT-3′; and *rpoD* RT-forward 5′-CTACAA GTCGAAGTCCTAC-3′ and RT-reverse 5′-ATCGACATCAGT TCGTTC-3′.

For the analysis of *plaI* and *plaR* gene expression, *B. plantarii* grown for 12 h in PDB containing carot-4-en-9,10-diol (20 µM), 3*-*methyl*-*2(5*H*)*-*furanone (50 µM) and DMDS (100 µM*)* were collected and subjected to isolation of total RNA and digestion of genomic DNA using NucleoSpin® RNA II kit (Macherey-Nagel, Düren, Germany). Purified RNA (1 µg) was utilized for synthesis of first strand cDNA with random 6 mers (5 µM, final concentration in a 10 µl-reaction system) using the PrimeScript™ II 1st Strand cDNA Synthesis Kit (Takara, Tokyo, Japan). Any remaining RNA was then removed by digestion with RNAse A (Nippon Gene, Toyama, Japan) at 37°C for 30 min. The resulting cDNA library was used as a template in combination with the specific primers for quantitative PCR, which was conducted using a SYBR Premix Ex Taq II Kit (Takara, Tokyo, Japan) in a thermal cycler real time dice (Takara TP800, Tokyo, Japan). Cycling was 30 s at 95°C; 5 s at 95°C, 30 s at 52°C, 1 min at 72°C, repeat for 40 cycles. Specificity of each PCR amplicon was assessed with the dissociation curve (15 s at 95°C, 30 s at 50°C, 15 s at 95°C). Each target gene was calculated and expressed as fold regulation in comparison to the housekeeping gene *rpoD* for each treatment according to the 2^−ΔΔ*C*T^ method [58], [59].

## Supporting Information

File S1 **Biofilm inducing effects of supplemental tropolone and carot-4-en-9,10-diol on** ***B. plantarii*** **cells.** Cell states of *B. plantarii* in PDB medium containing exogenous tropolone and/or carot-4-en-9,10-diol at 200 µM. *B. plantarii* grown in PDB containing neither exogenous tropolone nor carot-4-en-9,10-diol was control (A). The static culture of *B. plantarii* in PDB medium supplemented with 200 µM carot-4-en-9,10-diol (B), that cultured in 200 µM exogenous tropolone plus 200 µM carot-4-en-9,10-diol supplemented medium (C), and in 200 µM exogenous tropolone (D) (all shown in the left panels). For comparison of the biofilm formation induced by those chemical signals, biofilm biomass produced by *B. plantarii* cultured in PDB was evaluated crystal violet staining method to monitor at A_570_ (right panel E, all the chemicals treated were 200 µM). All the bacterial cultures were obtained after 48 h-incubation. Values are means ± SD (shown as error bars) (n = 3). *P<0.01 by Student’s-t test.
(PSD)

File S2 **Effects of tropolone and carot-4-en-9,10-diol on induction of biofilm biomass.** (A) Biofilm biomass was quantified from *B. plantarii* cultured for 24, 48, 72 and 96 h in PDB supplemented with iron (as FeCl_3_) tropolone at zero µM (blue diamond), 200 µM (red circle) and 500 µM (green triangle). (B) Biofilm biomass was quantified from B. plantarii cultured for 24, 48, 72 and 96 h in PDB supplemented with exogenous tropolone at zero µM (blue diamond), 100 µM (red circle) and 200 µM (green triangle). All the bacterial cultures were obtained after 48 h-incubation. Values are means ± SD (shown as error bars) (n = 3).
(PSD)

File S3 **Effect of quorum sensing inhibitors on tropolone production and B. plantarii cell growth.** (A) Cell density-dependent manner of tropolone production by *B. plantarii*. (B) Tropolone production (columns) and cell growth (plots) were quantified from *B. plantarii* PDB cultures containing 3-methyl-2(5*H*)-furanone at zero, 50, 100 and 200 µM. (C) Tropolone production (columns) and cell growth (plots) were quantified from *B. plantarii* PDB cultures containing dimethyl disulfide at zero, 50, 100 and 200 µM. Values are means ± SD (shown as error bars) (n = 3). *P<0.01 by Student’s-*t* test.
(PSD)

File S4 **^1^H-NMR spectrum of carot-4-en-9,10-diol.**
(PSD)

File S5 **^13^C-NMR spectrum of carot-4-en-9,10-diol.**
(PSD)
